# Investigating LETd optimization strategies in carbon ion radiotherapy for pancreatic cancer: a dosimetric study using an anthropomorphic phantom

**DOI:** 10.1002/mp.17569

**Published:** 2024-12-10

**Authors:** Filipa Baltazar, Friderike K. Longarino, Christina Stengl, Jakob Liermann, Stewart Mein, Jürgen Debus, Thomas Tessonnier, Andrea Mairani

**Affiliations:** ^1^ Heidelberg Ion‐Beam Therapy Center (HIT) Department of Radiation Oncology Heidelberg Germany; ^2^ Clinical Cooperation Unit Radiation Oncology German Cancer Research Center (DKFZ) Heidelberg Germany; ^3^ Medical Faculty Heidelberg University Heidelberg Germany; ^4^ Clinical Cooperation Unit Translational Radiation Oncology German Cancer Research Center (DKFZ) Heidelberg Germany; ^5^ Department of Radiation Oncology Heidelberg University Hospital Heidelberg Germany; ^6^ Division of Medical Physics in Radiation Oncology German Cancer Research Center (DKFZ) Heidelberg Germany; ^7^ Heidelberg Institute of Radiation Oncology (HIRO) Heidelberg Germany; ^8^ National Center for Tumor diseases (NCT) Heidelberg Germany; ^9^ Department of Accelerator and Medical Physics Institute for Quantum Medical Science National Institutes for Quantum Science and Technology Chiba Japan; ^10^ German Cancer Consortium (DKTK) Core Centre Heidelberg Heidelberg Germany; ^11^ Centro Nazionale di Adroterapia Oncologica (CNAO) Pavia Italy

**Keywords:** LETd‐boosting, pancreatic cancer, spot‐scanning hadron arc

## Abstract

**Background:**

Clinical carbon ion beams offer the potential to overcome hypoxia‐induced radioresistance in pancreatic tumors, due to their high dose‐averaged Linear Energy Transfer (LETd), as previous studies have linked a minimum LETd within the tumor to improved local control. Current clinical practices at the Heidelberg Ion‐Beam Therapy Center (HIT), which use two posterior beams, do not fully exploit the LETd advantage of carbon ions, as the high LETd is primarily focused on the beams’ distal edges. Different LETd‐boosting strategies, such as Spot‐scanning Hadron Arc (SHArc), could enhance LETd distribution by concentrating high‐LETd values in potential hypoxic tumor cores while sparing organs at risk.

**Purpose:**

This study aims to investigate and verify different LETd‐boosting strategies using an anthropomorphic pancreas phantom.

**Methods:**

Various LETd‐boosting strategies were investigated for a cylindrical and a pancreas‐shaped target in an anthropomorphic pancreas phantom. Treatment plans were optimized using single field optimization (SFO) or multi field optimization (MFO), with objective functions based on either physical dose (Phys), relative biological effectiveness (RBE)‐weighted dose, or a combination of RBE and LETd‐based objectives (LETopt). The LETd‐boosting planning strategies were optimized with the goal of increasing the minimum LETd in the tumor without compromising its homogeneous dose coverage. Beam configurations investigated included the two‐beam in‐house clinical standard (2‐SFO_Phys_, 2‐SFO_RBE_ and 2‐MFO_RBE‐LETopt_), a three‐beam configuration (3‐MFO_RBE_ and 3‐MFO_RBE‐LETopt_) and SHArc (SHArc_Phys_, SHArc_RBE_ and SHArc_RBE‐LETopt_) using step‐and‐shoot delivery. The different plans were verified using an anthropomorphic pancreas phantom at HIT and compared to treatment planning system (TPS) predictions.

**Results:**

All investigated LETd‐boosting strategies altered the LETd distribution while meeting optimization goals and constraints, resulting in varying degrees of LETd enhancement. For the cylindrical volume, the SHArc plan resulted in the highest LETd concentration in the tumor core, with the minimum LETd in the GTV scaling up to 91 keV/µm. For the pancreas‐shaped volume, however, the 3‐MFO_RBE‐LETopt_ achieved a higher minimum LETd in the GTV than SHArc_RBE_ (75.6 and 62.3 keV/µm, respectively). When combining SHArc with LETd optimization, a minimum LETd of 76.3 keV/µm was achieved, suggesting a potential benefit from this combined approach. Most dosimetric verifications showed dose deviations to the TPS within a 5% range, for both beam‐per‐beam and total dose. LETd‐optimized and SHArc plans exhibited slightly higher mean dose deviations (2.0%—4.6%) compared to the standard RBE‐based plans (<1.5%).

**Conclusion:**

This study demonstrated the feasibility of enhancing LETd in pancreatic tumors using carbon ion arc delivery coupled with LETd optimization. The possibility of delivering these plans was verified through irradiation of an anthropomorphic pancreas phantom, which showed agreement between dose measurements and predictions.

## 1 INTRODUCTION

Pancreatic cancer remains among the deadliest diseases, with a 5‐year survival rate of only 5%–10%.^1^, ^2^ Due to the diagnosis at late stage of disease, about one‐third of patients are diagnosed with locally advanced pancreatic cancer (LAPC).^3^ Compared to other sites, pancreatic tumors often exhibit severe hypoxia, with oxygen levels below 2.5 mmHg,^4^ making them highly resistant to conventional photon‐based radiotherapy (RT).

Carbon Ion Radiotherapy (CIRT) stands out for its superior relative biological effectiveness (RBE) and higher Linear Energy Transfer (LET), which reduce RT sensitivity to changes in the tumor's oxygenation, therefore making it potentially more effective against hypoxia‐induced radioresistance.^5^ To date, most clinical studies on CIRT for pancreatic cancer have been conducted in Japanese clinical centers, which have shown superior outcomes compared to published photon data with a median survival time of 25.1 months for 64 patients.^6^ Hagiwara et al. correlated a minimum dose‐averaged Linear Energy Transfer (LETd) in the Gross Tumor Volume (GTV) of 44 keV/µm with an improved Local Control (LC).^7^

The first European phase II clinical trial, the PACK trial, is currently taking place at the Heidelberg Ion‐Beam Therapy Center (HIT).^8^ Previous studies indicated that HIT's clinical standards align with the Japanese standards in terms of tumor's dose coverage.^9^ However, in terms of LETd, less than 15% of patients exhibited a minimum LETd in the GTV exceeding 44 keV/µm. Additionally, given the two‐posterior beam configuration used for patient irradiation in the PACK trial, high‐LETd values are primarily concentrated at the distal edge of the tumor.

Strategies to boost LETd in the tumor volume have been widely investigated, with the first clinical trial investigating LETd painting with CIRT reporting its safety and efficacy in a cohort of 12 head and neck cancer patients.^10^ Another strategy involves multi‐ion optimization, where both lower and higher LET particle beams are combined in a single treatment session.^11^ However, the limited availability of centers worldwide capable of delivering ions heavier than carbon ions restricts the clinical application of multi‐ion optimization to a few specialized centers and patients.^11^, ^12^, ^13^ Additionally, in silico‐studies for Simultaneously Integrated Boost (SIB), in which a tumor's subregion receives a dose boost, have also shown potential in increasing the LETd in the GTV for patients with LAPC.^14^ Furthermore, ongoing clinical trials at the Shanghai Proton and Heavy Ion Center are evaluating SIB's efficacy specifically for pancreatic cancer treatment.^15^

Finally, there has been a growing interest in novel treatment delivery techniques such as particle arc therapy. In‐silico studies by Mein et al. demonstrated Spot‐scanning Hadron Arc (SHArc) therapy could deliver a conformal dose to the tumor, while sparing the surrounding normal tissues, which are irradiated with lower dose levels but in higher volume (the so‐called low‐dose bath).^16^, ^17^, ^18^ The arc‐irradiation improves the LETd distribution, focusing the high LETd towards the tumor's hypoxic core. Furthermore, recent work by Tessonnier et al.^19^ reported the first SHArc delivery and dosimetric verification with carbon ions using a cylindrical phantom, providing experimental evidence in vitro that supports SHArc's potential role in overcoming hypoxia‐induced radioresistance. In this work, the first dosimetric verification of different LETd boosting strategies, for example, SHArc, is performed using an anthropomorphic pancreas phantom to evaluate clinical feasibility and identify challenges associated with SHArc delivery for pancreatic tumors.

## 2 METHODS

### 2.1 Experimental setup

**FIGURE 1:**
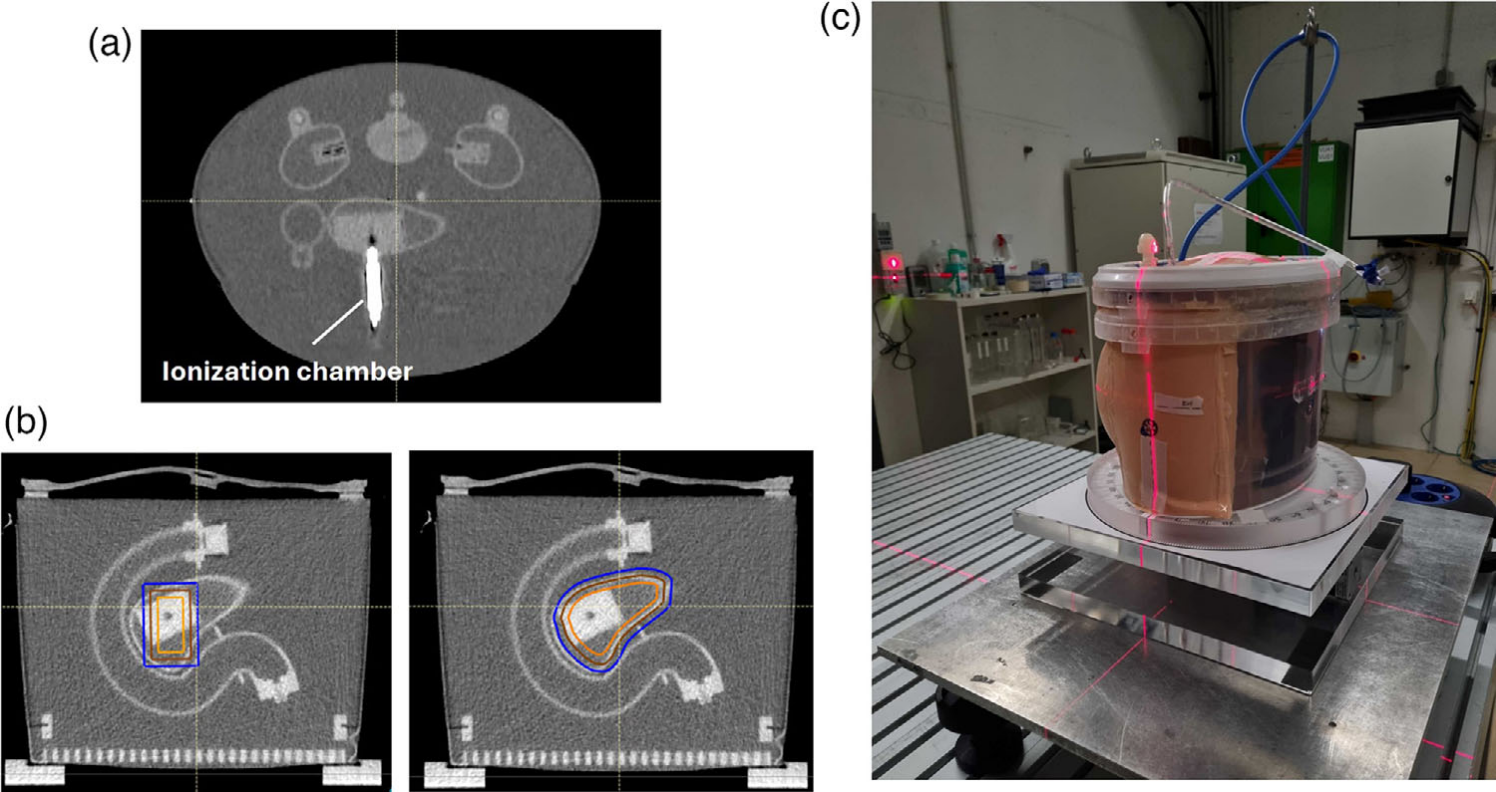
(a) Axial CT slice of the pancreas phantom for ion‐beam therapy (PPIeT) phantom containing the PinPoint‐TM31015 chamber (PTW), in white, positioned in the pancreas, (b) planning target volumes (PTV) in this study (in blue): a cylinder and a pancreas‐shaped PTV (left and right, respectively). Additionally, the clinical target volume (CTV) and gross tumor volume (GTV) are shown (in brown and orange, respectively). (c) Experimental setup for plan irradiation: PPIeT phantom positioned on the rotational stage.

An anthropomorphic pancreas phantom (Pancreas Phantom for Ion‐beam Therapy, PPIeT) developed by Stengl et al.^20^ was used for the treatment planning and verification carried out in this study. This phantom includes a 3D‐printed pancreas and two kidneys, both filled with agarose‐based mixtures, a duodenum, a spine, and a spinal cord. Further details regarding the phantom can be found in its original publication.^20^ Additionally, the pancreas contains a pluggable insert where an ionization chamber (PinPoint‐TM31015 chamber, PTW) can be placed for dosimetric measurements (Figure 1a).

### 2.2 CT image acquisitions of the phantom

CT images of the PPIeT phantom were obtained prior to irradiation on a SOMATOM Confidence CT scanner (Siemens Healthineers, Forchheim, Germany) using a sequential acquisition dual‐energy computed tomography (DECT) technique. DECT has been shown to reduce range uncertainties because it allows the uncertainty arising from the generalized conversion between CT numbers and the stopping power ratio (SPR) in single‐energy CT (SECT) to be mitigated.^21^, ^22^, ^23^ The DECT data was employed as the planning CT, thereby facilitating enhanced accuracy for particle range estimation.^24^ For SPR prediction from the DECT image data, a DirectSPR implementation^25^ was used in the syngo.via image reconstruction software (Siemens Healthineers, Forchheim, Germany). Following this optimization, forward plan calculations were performed on SECT image data to assess differences in the planned dose distribution. The 140 kV_p_ image data were used for SECT‐based SPR prediction using a Hounsfield look‐up table (HLUT).^26^, ^27^, ^28^

On the day of irradiation, an additional DECT (daily CT) was acquired, which permitted the accounting for changes in the position of the measurement point. These adjustments were integrated into the TPS estimates and in the respective deviations’ calculations. Further details regarding this correction can be found in the Supplementary Material (S1).

### 2.3 Treatment planning strategies

The different optimization strategies using carbon ions investigated in this work are summarized in Table 1. All treatment plans were optimized using the RayStation 2024A‐DTK treatment planning system (TPS) (RaySearch Laboratories), considering objective functions based on physical dose (Phys), RBE‐weighted dose (RBE), or a combination of RBE and LETd‐based objectives (LETopt), with LETd calculated as reported in work by Fredriksson et al.^29^ Plans were optimized using three beam configurations: a 2‐beam, 3‐beam and SHArc step‐and‐shoot configuration. All plans were verified with an anthropomorphic pancreas phantom at the Heidelberg Ion‐Beam Therapy Center (HIT; Heidelberg, Germany)^30^ and compared to TPS predictions.

**TABLE 1:** Summary of planning strategies and corresponding dosimetric measurements. Plans were optimized using single field optimization (SFO) or multi‐field optimization (MFO) for 2‐beam, 3‐beam, and spot‐scanning hadron arc (SHArc) configurations, based on physical dose (Phys), RBE‐weighted dose (RBE), or LETd‐based objective functions (LETopt). The table lists near‐minimum and near‐maximum dose‐averaged LET (keV/µm) in the GTV and the expected physical dose (Gy) at the point of measurement computed in the TPS. Additionally, the mean measured dose following three irradiations (Gy), with standard deviation (%), as well as the mean deviation from expected dose (%), with standard deviation (%), are reported.

Target (volume)	Plan	Beam configuration	Objective functions	GTV LETd_98%_ (keV/µm, TPS)	GTV LETd_2%_ (keV/µm, TPS)	TPS dose (Gy)	Measured dose (Gy)	Deviation to TPS (%)
Cylinder [12.36 cm^3^]	2‐SFO_Phys_	2 Beams (posterior)	Physical dose objectives	44.5	61.6	4.01	3.99 (±0.10%)	0.47 (±0.07%)
	2‐SFO_RBE_		RBE weighted‐dose objectives	49.1	66.6	1.87	1.86 (±0.48%)	0.38 (±0.19%)
	2‐MFO_RBE‐LETopt_		RBE weighted‐dose and LETd‐based objectives	73.1	81.1	1.72	1.79 (±1.17%)	4.04 (±0.83%)
	3‐MFO_RBE_	3 Beams (2posterior+1anterior)	RBE weighted‐dose objectives	42.0	54.4	1.85	1.85 (±0.11%)	0.15 (±0.10%)
	3‐MFO_RBE‐LETopt_		RBE weighted‐dose and LETd‐based objectives	75.3	85.3	1.73	1.79 (±0.00%)	3.22 (±0.02%)
	SHArc_Phys_	SHArc (20 beams: spaced 18° in a 360° arc)	Physical dose objectives	90.4	102.6	4.02	4.10 (±0.34%)	1.97 (±0.33%)
	SHArc_RBE_		RBE weighted‐dose objectives	91.2	103.0	1.70	1.74 (±1.09%)	2.66 (±1.37%)
Pancreas [27.41 cm^3^]	2‐SFO_Phys_	2 Beams (posterior)	Physical dose objectives	43.0	59.9	4.00	3.98 (±0.08%)	0.42 (±0.07%)
	2‐SFO_RBE_		RBE weighted‐dose objectives	48.6	68.8	1.84	1.84 (±0.16%)	0.13 (±0.14%)
	2‐MFO_RBE‐LETopt_		RBE weighted‐dose and LETd‐based objectives	63.5	73.0	1.79	1.86 (±0.11%)	4.05 (±0.09%)
	3‐MFO_RBE_	3 Beams (2posterior+1anterior)	RBE weighted‐dose objectives	43.8	53.6	1.85	1.84 (±1.79%)	1.56 (±0.83%)
	3‐MFO_RBE‐LETopt_		RBE weighted‐dose and LETd‐based objectives	75.6	84.1	1.78	1.81 (±1.00%)	2.57 (±1.51%)
	SHArc_Phys_	SHArc (20 Beams: spaced 18° in a 360° arc)	Physical dose objectives	63.1	101.4	4.02	4.15 (±0.79%)	3.16 (±0.78%)
	SHArc_RBE_		RBE weighted‐dose objectives	62.3	101.7	1.68	1.76 (±1.37%)	4.15 (±1.34%)
	SHArc_RBE‐LETopt_		RBE weighted‐dose and LETd‐based objectives	76.3	101.3	1.66	1.75 (±0.29%)	4.60 (±0.27%)

The 2‐SFO_Phys_ and 2‐SFO_RBE_ plans were optimized using Single Field Optimization (SFO) to achieve a uniform dose distribution per beam. In contrast, the remaining plans, 2‐MFO, 3‐MFO and SHArc plans, were optimized using Multi Field Optimization (MFO), for a homogeneous final dose distribution, regardless of the individual distribution of each field.

Two different volumes were delineated and considered as Planning Target Volume (PTV): a simpler target consisting of a cylinder with a 2 cm radius and 6 cm height, and a second PTV, representing a more clinically relevant volume, consisting of the head of the pancreas in the phantom (as illustrated in Figure 1b). For each PTV, a Clinical Target Volume (CTV) was derived by contracting the PTV by 5 mm in all directions, followed by deriving the GTV through a further 5 mm contraction of the CTV. Thus, for each of the plans ‐ 2‐SFO_Phys,_ 2‐SFO_RBE,_ 2‐MFO_RBE‐LETopt,_ 3‐MFO_RBE,_ 3‐MFO_RBE‐LETopt,_ SHArc_Phys_ and SHArc_RBE—_two distinct plans were created: one for the cylindrical PTV and one for the pancreas‐shaped PTV. Additionally, for the pancreas‐shaped PTV, an additional SHArc_RBE‐LETopt_ plan, was created (resulting in a total of 15 plans).

Three different beam configurations were studied in this work. Initially, the clinical standard beam configuration used at HIT for pancreatic cancer treatment was considered, which consists of 2 posterior beams (separated by 32° in our setup). Secondly, a configuration with three beams was studied, in which a third anterior beam was added to the initial two‐beam configuration, aiming at a rearrangement of LETd's spatial distribution in the PTV. Finally, SHArc plans were optimized for step‐and‐shoot delivery, using 20 beams spaced equally along a 360° arc (i.e., every 18°). To limit irradiation time and enhance plan robustness, each beam in the SHArc plan was restricted to its 9 central energy layers. For this, a pre‐optimization step was performed, in which spots were initially positioned in the tumor according to the beam configuration and tumor volume definition, without limiting the number of energy layers per beam. Subsequently, only the nine central energy layers, for example, central to the PTV volume, were retained for further optimization.

Three main plan categories can be distinguished based on the objective functions used for plan optimization. Firstly, physically optimized plans, such as 2‐SFO_Phys_ and SHArc_Phys,_ were optimized using only physical dose objectives, to deliver a uniform physical dose of 4 Gy in the PTV. This first approach aimed to minimize physical dose gradients within the tumor. Secondly, biologically optimized plans (such as in 2‐SFO_RBE,_ 3‐MFO_RBE_ and SHArc_RBE_) were optimized using RBE‐weighted dose objectives. Similarly to the clinical standard at HIT for pancreatic cancer patients, the Local Effect Model (LEM‐I) was used for RBE calculations, with a differential (α/β) assigned for tumor and healthy tissue of 5 and 2 Gy respectively. For these plans, 4 Gy(RBE) was prescribed to the PTV. For both physically and biologically optimized plans, no additional dose objectives were considered for the organs at risk (OARs). Finally, for the 2‐MFO_RBE‐LETopt,_ 3‐MFO_RBE‐LETopt_ and SHArc_RBE‐LETopt_ plans, additional minimum LETd‐based objectives for the tumor region were included in the optimization, using the functions available in the current research version of RayStation. For most LETd‐plans, a minimum LETd of 65 and 75 kev/µm in the CTV and GTV was added as objective function. For cases in which these LETd‐objectives compromised the CTV's homogeneous dose coverage (Homogeneity Index, HI = (*D*_2%_ − *D*_98%_)/*D*_50%,_ above 0.05), the LETd objective functions were adjusted to 60 and 65 kev/µm, for CTV and GTV respectively. While a SHArc_RBE‐LETopt|pancreas_ plan was created specifically for the pancreas‐shaped PTV, a similar plan combining SHArc with LETd optimization was found unnecessary for the cylindrical PTV, as, for this case, the SHArc_RBE|cylinder_ plan already provided superior minimum LETd coverage for the cylindrical PTV compared to other plans.

All plans were optimized using the following beam specifications: minimum beam width in air at the isocenter of 6 mm, following a hexagonal spot pattern with a 2.4 mm spot spacing, and a 3.0 mm energy layer spacing. Additionally, the beams were filtered to retain the minimum number of particles per spot delivered, compatible with the HIT delivery system (1.5 × 10^4^ for carbon ions). Total delivery time, including both plan irradiation and stage rotation between angles, ranged from 5 min for the 2‐beam plans to approximately 20 min for the SHArc plans.

### 2.4 Plan evaluation

Throughout this work, the different plans were evaluated using metrics such as *D_x_*_%_, where “*x*” represents a specific percentage of a target volume or organ at risk. For example, *D*_50%_ indicates the dose received in at least 50% of the volume. Similarly, metrics for the LETd are used, which measure the LETd for the most exposed *x*% of the volume.

Additionally, a robust evaluation of the plans was performed by simulating range errors of ±2% and setup shifts of up to 2 mm in all directions, resulting in a total of 21 different scenarios. The following clinical constraints were assessed: *D*_95%_ in the CTV should be greater than 95% of the prescribed dose, and *D*_1%_ in the CTV should be less than 105% of the prescribed dose. This evaluation was only performed for plans considering RBE‐ and/or LETd‐based objective functions in the optimization.

### 2.5 Plan irradiation

Each optimized plan was irradiated three times at HIT. For this, the phantom was positioned on a rotating stage equipped with a motor capable of a minimum rotating step of 2.25° (MM Engineering) (Figure 1c). For the SHArc plans, a step‐and‐shoot delivery was used, with the rotating stage synchronized to rotate after the last energy layer of each beam, in coordination with the accelerator. This synchronization ensured that the rotating stage could complete an 18° rotation before the next beam was irradiated, which took approximately 18 s.

Following plan irradiation, the deviations between the measured and the TPS values were computed according to the quality assurance (QA) procedure of HIT,^31^ following Equation (1):

(1) $$\mathrm{Deviation}=\frac{\left|D_{\mathrm{meas}}-D_{\mathrm{TPS}|50\%}\right|}{D_{\mathrm{TPS}|\max}}\times 100\left(\%\right)$$

In which *D*_meas_ is the measured physical dose on the day of irradiation, and *D*_TPS|max_ and *D*_TPS|50%_ are the plan's maximum dose and the median physical dose at the point of measurement computed by the TPS, respectively.

## 3 RESULTS

### 3.1 Treatment planning strategies

**FIGURE 2:**
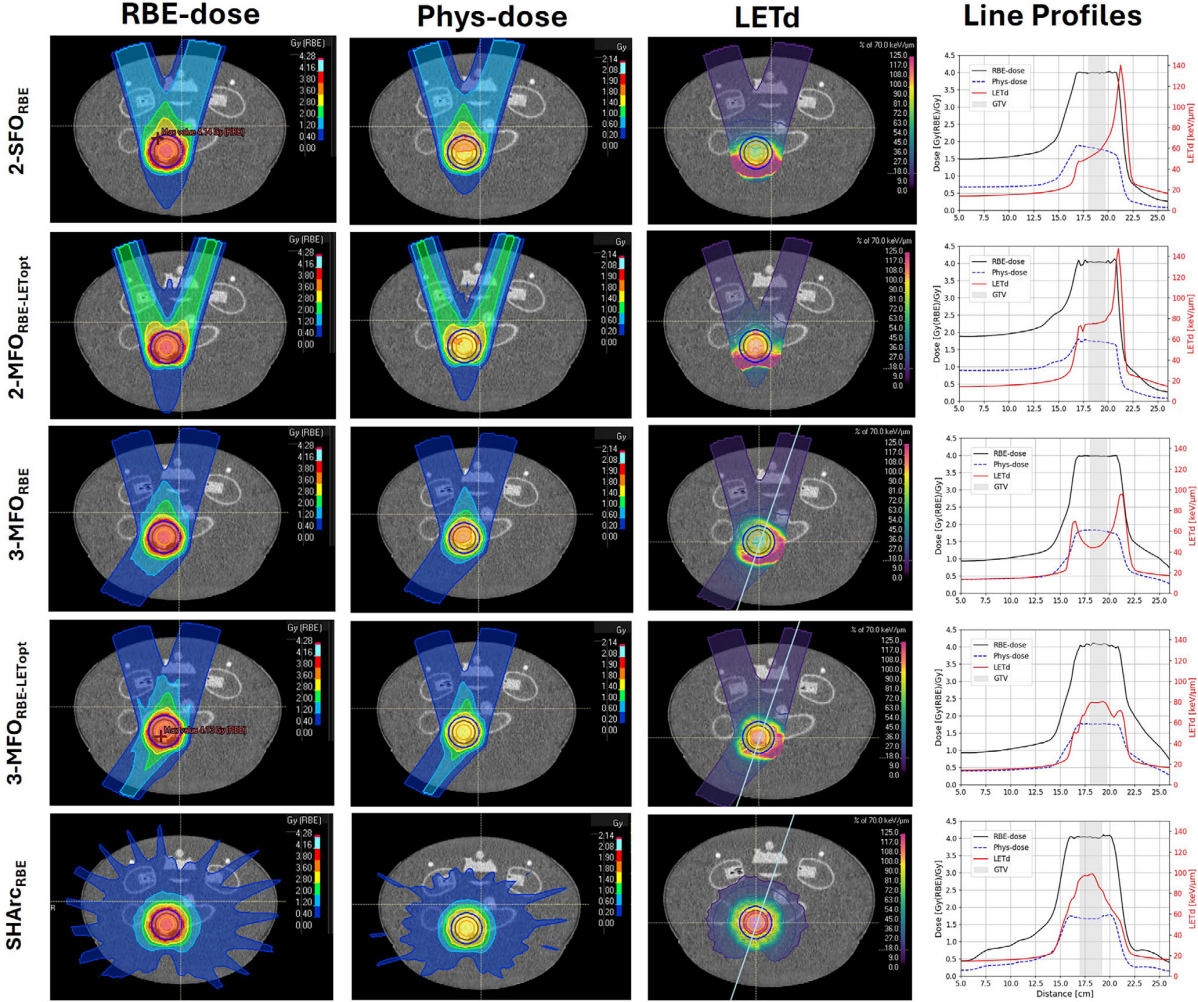
Treatment planning strategies implemented for the cylindrical target. For each plan (line in the figure): axial slice of the relative biological effectiveness (RBE)‐weighted dose and the physical dose distributions, as well as dose‐averaged linear energy transfer (LETd) distribution (left to right). Moreover, RBE‐weighted and physical dose profiles (in black and blue), as well as LETd profiles (in red) across a line (in white) are represented as a function of penetrated depth (in cm).

**FIGURE 3:**
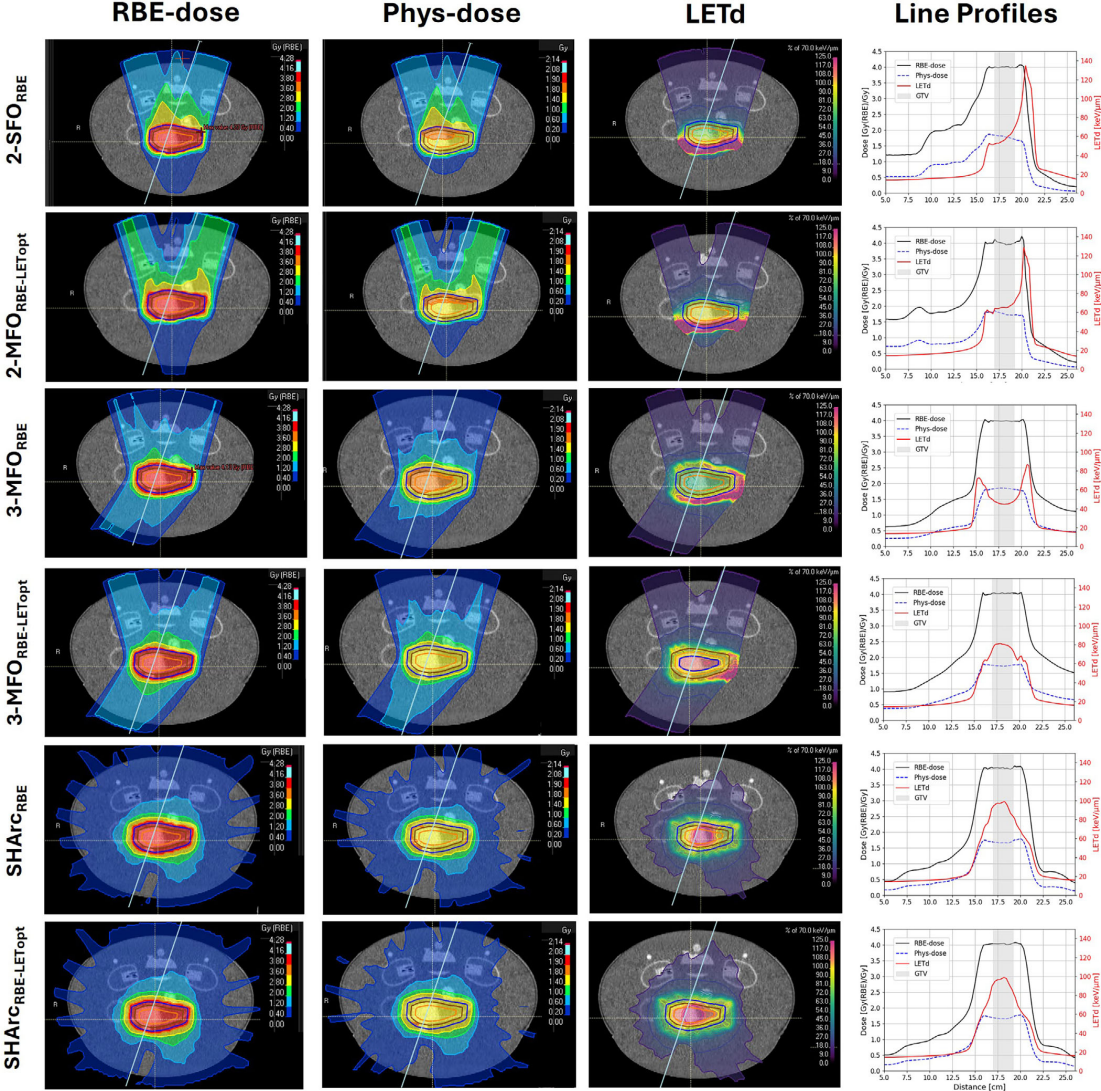
Treatment planning strategies implemented for the pancreas‐shaped target. For each plan (line in the figure): axial slice of the relative biological effectiveness (RBE)‐weighted dose and the physical dose distributions, as well as dose‐averaged linear energy transfer (LETd) distribution (left to right). Moreover, RBE‐weighted and physical dose profiles (in black and blue), as well as LETd profiles (in red) across a line (in white) are represented as a function of penetrated depth (in cm).

Figures 2 and 3 show axial slices of the RBE‐dose, physical dose, and LETd distributions of the RBE‐ and LETd‐optimized plans for the cylindrical and the pancreas‐shaped PTV, respectively. The physically optimized plans, along with additional dose profiles for the various beam setups and optimization strategies considered in this work, can be found in the Supplementary Material (S2). The near‐minimum and near‐maximum LETd (LETd_98%_ and LETd_2%_, respectively) in the GTV for all plans are also reported in Table 1.

As shown in the figures, when two beams were used for plan optimization, the high‐LETd component focused on the tumor's distal edge. When LETd optimization was combined with this beam configuration, an increase in LETd_98%_ from 49.1 keV/µm to 73.1 keV/µm was observed for the cylindrical target. For the larger pancreas‐shaped target, the LETd boost observed was smaller, with LETd_98%_ increasing from 48.6 keV/µm to 63.5 keV/µm. Additionally, for the two‐beam configuration, LETd‐optimization also led to a higher entrance dose, with one beam's entrance dose rising from 1.5 Gy(RBE) in the 2‐SFO_RBE|cylinder_ plan to 2 Gy(RBE) in the 2‐MFO_RBE‐LETopt|cylinder_ plan, as shown in Figure 2. Similar results were observed for the pancreas‐shaped PTV, as shown in Figure 3.

Introducing a third beam generally reduced overall LETd values, with LETd_98%_ decreasing to 42 keV/µm and 43.8 keV/µm for the 3‐MFO_RBE|cylinder_ and 3‐MFO_RBE|pancreas_, respectively. However, the additional beam allowed a higher LETd boost without compromising tumor dose coverage. Specifically, the combination of LETd optimization with the 3‐beam configuration led to a minimum LETd of 75 keV/µm for both PTV volumes.

The SHArc plans effectively provided conformal dose coverage while concentrating the high‐LETd component at the tumor's central, often hypoxic, core. For the cylindrical target, the SHArc plan achieved a LETd_98%_ of 91.2 keV/µm. However, for the larger pancreas target, the LETd_98%_ achieved was lower, at 62.3 keV/µm. Thus, for this volume, the minimum LETd obtained with the SHArc plan was lower than the minimum LETd obtained with the respective 3‐beam LETd‐optimized plan. To address this, an additional SHArc_RBE‐LETopt_ plan was created, combining SHArc with LETd optimization, which increased the LETd_98%_ to 76.3 keV/µm.

### 3.2 Robust evaluation

The robust evaluation of optimization strategies for the cylinder and pancreas PTV showed varied robustness in meeting dose criteria, as shown in Table 2. The 2‐SFO_RBE_ and 3‐MFO_RBE_ strategies consistently passed 100% of scenarios with D_95%|CTV _> 3.8 Gy(RBE) and D_1%|CTV _< 4.2 Gy(RBE), for both target volumes. However, strategies including LETd‐optimization and SHArc plans resulted in a less robust passed rate, with 57% to 67% of scenarios meeting the criteria, and worst‐case D_1%|CTV_ values reaching up to 4.50 Gy(RBE) in the case of the SHArc plans. Moreover, two distinct trends were observed in the robust evaluation of the SHArc plans. When a range shift of +2% was simulated, a cold spot developed in the center of the tumor, leading to a failure in 1/3 of scenarios concerning the minimum CTV coverage criteria. Conversely, a ‐2% range shift resulted in a hotspot within the CTV, causing 1/3 of scenarios to fail in meeting the maximum dose limit for the CTV.

**TABLE 2:** Robust evaluation of optimization strategies for RBE‐ and LETd‐based plans, showing the percentage of scenarios meeting D_95%|CTV_ ≥ 3.8 Gy (RBE) and D_1%|CTV_ ≤ 4.2 Gy (RBE) constraints, and the worst‐case scenario doses for cylindrical and pancreatic targets.

		D_95%|CTV _> 3.8 Gy (RBE)		D_1%|CTV _< 4.2 Gy (RBE)	
Optimization strategies		Number of passed scenarios [%]	Worst case scenario [Gy(RBE)]	Number of passed scenarios [%]	Worst case scenario [Gy(RBE)]
Cylinder	2‐SFO_RBE_	100	3.92	100	4.07
	2‐MFO_RBE‐LETopt_	67	3.60	67	4.44
	3‐MFO_RBE_	100	3.84	100	4.15
	3‐MFO_RBE‐LETopt_	67	3.69	67	4.33
	SHArc_RBE_	67	3.54	57	4.50
Pancreas	2‐SFO_RBE_	100	3.90	100	4.20
	2‐MFO_RBE‐LETopt_	95	3.77	95	4.24
	3‐MFO_RBE_	100	3.87	100	4.12
	3‐MFO_RBE‐LETopt_	67	3.69	67	4.32
	SHArc_RBE_	67	3.62	62	4.5
	SHArc_RBE‐LETopt_	67	3.62	62	4.49

### 3.3 Comparison between DECT and SECT

Although all treatment plans were optimized using DECT images for their superior range prediction accuracy, forward calculations were also performed using SECT images to assess any potential discrepancies. The percentage ΔD_1%_ and ΔD_99%_ in the PTV between DECT and SECT are detailed in Table S3.1 of the Supplementary Material (S3). Overall, discrepancies are more pronounced for D_1%_ than for D_99%_. Additionally, SHArc plans exhibit larger differences compared to the 2‐ and 3‐field configurations. Specifically, for the physical SHArc plans, the maximum deviations are approximately 1.8% for ΔD_1%_ and 4% for ΔD_99%_, compared to 0.5% and 1.5%, respectively, for the other plans.

### 3.4 Dosimetric verifications

The expected physical dose at the point of measurement, as well as the mean measured dose and the respective mean deviation (as per Equation 1) are provided for each plan in Table 1. Additional details on deviation calculations and beam‐by‐beam dose measurements are provided in the Supplementary Material (S4).

While the dosimetric deviations across the various treatment plans reveal varying levels of agreement with the TPS, all deviations to the expected physical dose remained within 5%, therefore indicating the overall feasibility of the studied strategies. For both cylindrical and pancreas‐shaped volumes, the 2‐SFO_Phys_ and 2‐SFO_RBE_ plans consistently demonstrated low mean deviations to the dose on the TPS, smaller than 0.5%. This indicates high agreement between the TPS predictions and the measured values. When LETd optimization was combined with the 2‐beam configuration, an increase in the mean deviations was observed, with the 2‐MFO_LETd_ plans showing a mean deviation to the TPS of approximately 4% to both cylinder and pancreas plans. The same behavior was observed for 3‐beam configuration, with the 3‐MFO_RBE‐LETopt_ plans exhibiting higher mean deviations to the TPS in comparison to the initial 3‐MFO_RBE_ plans. Finally, for both SHArc_Phys_ plans, the measured dose deviated by 2–3% from the expected dose. These deviations were higher than those observed in the 2‐beam physical plans, highlighting the increased complexity of the SHArc approach. When the plans were optimized using RBE‐weighted dose and LETd‐based objectives, the deviations increased further. Specifically, for the pancreas plans, approximate deviations of 4.15% and 4.60% were observed for the SHArc_RBE_ and SHArc_RBE‐LETopt_ plans, respectively.

## 4 DISCUSSION

In this study, we investigated the effects of LETd optimization and SHArc delivery on both the LETd distribution and plan robustness, as tested through irradiation of these plans in an anthropomorphic pancreas phantom (PPIeT). Initially, all plans were optimized to deliver a uniform physical or RBE‐weighted dose to the PTV, targeting either a cylindrical or pancreas‐shaped volume. In addition, LETd objectives were incorporated into the 2‐MFO_RBE‐LETopt_, 3‐MFO_RBE‐LETopt_ and SHArc_RBE‐LETopt_ plans.

Combining LETd optimization with the 2‐beam configuration allowed to increase the LETd within the tumor, by adjusting the relative weights of energy layers, favoring lower energies to shift the Bragg peaks inside the tumor. However, not only was this boost in LETd limited by the beam configuration, but it also came with a trade‐off of higher entrance dose, which can also potentially impact normal tissue toxicity.

In this regard, compared to the initial 2‐beam set‐up, the 3‐beam configuration allowed for greater LETd boost without compromising tumor coverage, particularly for the largest PTV. Additionally, the LETd‐optimized plans using 3 beams showed reduced increase in entrance dose compared to the 2‐beam LETd‐optimized plans. However, the 3‐beam configuration is not commonly used in clinical pancreatic treatments due to its increased sensitivity to anatomical changes, particularly due to filling changes in the gastrointestinal tract and the respiratory movement.

SHArc plans concentrated the high‐LETd at the tumor's core, which could potentially benefit the treatment of hypoxic regions typically found in pancreatic tumors. For the cylindrical target, SHArc plans resulted in higher LETd values in the tumor than the remaining planning strategies studied. For the larger pancreas‐shaped target, however, the 3‐beam LETd‐optimized plan led to higher minimum LETd in the GTV than the SHArc plan. In this context, combining SHArc with LETd optimization could further enhance LETd in the tumor, suggesting a potential advantage of this combined approach for specific cases.

Pre‐selecting the nine central energy layers for each beam in the SHArc configuration was key to speeding up delivery times while keeping the high‐LETd focus at the tumor center, achieving LETd_2%_ in the GTV of up to 100 keV/µm. Irradiation times ranged from 5 to 20 min depending on the number of beams, but this variation did not affect the dosimetric verification results.

To further assess the plans robustness, a robust evaluation in which range errors of ± 2% and setup shifts of up to 2 mm in all directions were simulated (resulting in a total of 21 scenarios) Overall, the robustness evaluation showed that while the 2‐SFO_RBE_ and 3‐MFO_RBE_ strategies consistently met dose criteria in terms of minimum target coverage, LETd‐optimized and SHArc plans were less robust, particularly with range uncertainties leading to large deviations in dose.

This reduced robustness was further reflected in the dosimetric verification, where LETd optimization and SHArc plans showed higher deviations between expected and measured doses. Despite this, most plans‐maintained mean and maximum deviations within 5% of the expected TPS doses, in terms of total and beam‐by‐beam dose. Only the 3‐MFO_RBE‐LETopt|pancreas_ plan exhibited a larger deviation, approaching 10% for one beam, which could possibly be attributed to the steep dose gradient in the region of the pinpoint chamber (see Figure S2.2 in the Supplementary Material). Nonetheless, it's worth noting that also for this plan, the total dose deviation remained within 5%, demonstrating overall good performance.

The impact of LETd optimization on plan robustness observed in this work aligns with the concept of the “LET trilemma”, which highlights a conflict between achieving high LETd and a homogeneous dose coverage in the target and maintaining robustness against range uncertainties.^29^ As previous studies have suggested, robust optimization may therefore be essential for LETd‐boosting strategies like arc therapy,^32^ emphasizing the need for dedicated arc optimization algorithms that balance delivery efficiency, plan quality, and robustness—or, alternatively, for exploring multi‐ion therapy strategies to help mitigating these uncertainties.^18^ Further, for potential clinical implementation, it will be important to assess inter‐fractional robustness, as anatomical variations may have an impact on the goals of the optimization strategies (both for dose and LETd coverage) and thus affect treatment outcome.

Despite challenges in dose coverage, one could hypothesize that increasing LETd within the tumor may outweigh the reductions in dose homogeneity, given its potential correlation with enhanced local tumor control. Therefore, to achieve a more balanced assessment of the trade‐off between reduced physical robustness (i.e. dose coverage) and the possible benefits of increased LETd, robust evaluation should extend beyond the impact that positional and density shifts have in the RBE‐weighted dose, to also include the effects of LETd boosting on biological effectiveness.

This study successfully conducted dosimetric verifications for LETd‐optimized and SHArc plans using an anthropomorphic pancreas phantom. Particularly, a single point measurement was performed, which could be in regions of high physical gradient for each beam. In future work towards clinical implementation, this high dose gradients could potentially raise challenges in clinical QA such as at HIT, in which 24 ionization chambers (IC) positioned in a water phantom are used for plan verification. To enhance Patient‐Specific Quality Assurance (PSQA) for routine SHArc use, employing a 2D IC Array could provide a comprehensive 2D dose distribution independent of the TPS. However, with the current gantry settings at HIT, verifying a SHArc plan with 20 beams and 9 energy layers could take up to 40 min due to the gantry's rotation speed, isoenergy layer changes, and record saving in‐between beams. In this aspect, the possibility to employ multi‐energy extraction, which allows for the delivery of more than one isoenergy within the same spill, could contribute to accelerating this process.^33^, ^34^

Future work incorporating LETd measurements into PSQA could improve patient treatment. Due to the sensitivity limits of the silicon‐based microdosimeters used in their study for plan verification, Koto et al. set a maximum target LETd_min_ of 70 keV/µm for the LETd‐optimized plans performed for head and neck patients using CIRT.^10^ Given that our study involves LETd values up to 100 keV/µm, this threshold is insufficient, suggesting a need for different measurement approaches. In this context, diamond detectors, as demonstrated by Magrin et al.,^35^ offer promising potential for microdosimetry in clinical carbon ion beams and could be a valuable direction for future research and clinical application.

Finally, incorporating Monte Carlo (MC) simulations as an independent check could improve the quality assessment of various treatment plans. Although these simulations are computationally more intensive, they provide a more accurate estimate of dose distribution compared to the analytical algorithms used in conventional TPS.^36^ Moreover, when combined with machine log files, MC simulations can also help reconstruct detailed dose and LETd maps based on actual delivery conditions.^37^, ^38^ Thus, future work towards incorporating in‐silico QA in the plan verification routine could further enhance the treatment's precision and reliability, addressing the complexities introduced by LETd‐optimization strategies in the dose profiles.

## 5 CONCLUSIONS

This study demonstrated the feasibility of enhancing LETd in pancreatic tumors using LETd‐boosting strategies and performed the first dosimetric verification for SHArc step‐and‐shoot delivery using an anthropomorphic pancreas phantom. Although these methods showed promising increases in LETd, they also presented some challenges with robustness and verification accuracy. Nonetheless, deviations between the dosimetric measurements and the expected value remained within acceptable limits. Future work should focus on refining QA protocols and verification methods to further advance these strategies.

## CONFLICT OF INTEREST STATEMENT

J.L. received travel reimbursement from RaySearch Laboratories AB and Micropos Medical outside the submitted work. The remaining authors have no relevant conflicts of interest to disclose.

## Supporting information



Supporting information
