# Supplementary material for: Investigating LETd optimization strategies in carbon ion radiotherapy for pancreatic cancer: a dosimetric study using an anthropomorphic phantom
Source: Med Phys. 2024 Dec 10;52(3):1746–57. doi: 10.1002/mp.17569 (PMC11880654; doi:10.1002/mp.17569)
Supplement: Supplementary file 1 — Supporting information [file MP-52-1746-s001.pdf]

Investigating LETd optimization strategies in carbon ion radiotherapy for pancreatic cancer: a dosimetric study using an anthropomorphic phantom

Supplementary Material

S1. CT image on irradiation day ("daily CT")

CT images of the PPIeT phantom were obtained prior to irradiation on a SOMATOM Confidence CT scanner (Siemens Healthineers, Forchheim, Germany) using a sequential acquisition dual-energy computed tomography (DECT) technique. The following image acquisition settings and reconstruction parameters were used: tube voltage of 80/140 kV_p, tube current-time product of 73/42 mAs using tube-current modulation, collimation of 2 × 32 × 0.6 mm, rotation time of 0.5 s, pitch of 0.6/1.2, $CTDI_{vol}$ of 3.0/4.2 mGy using 32 cm $CTDI_{vol}$ diameter, slice thickness and slice spacing of 1.5 mm, and Qr40 reconstruction filter with bone beam hardening correction.

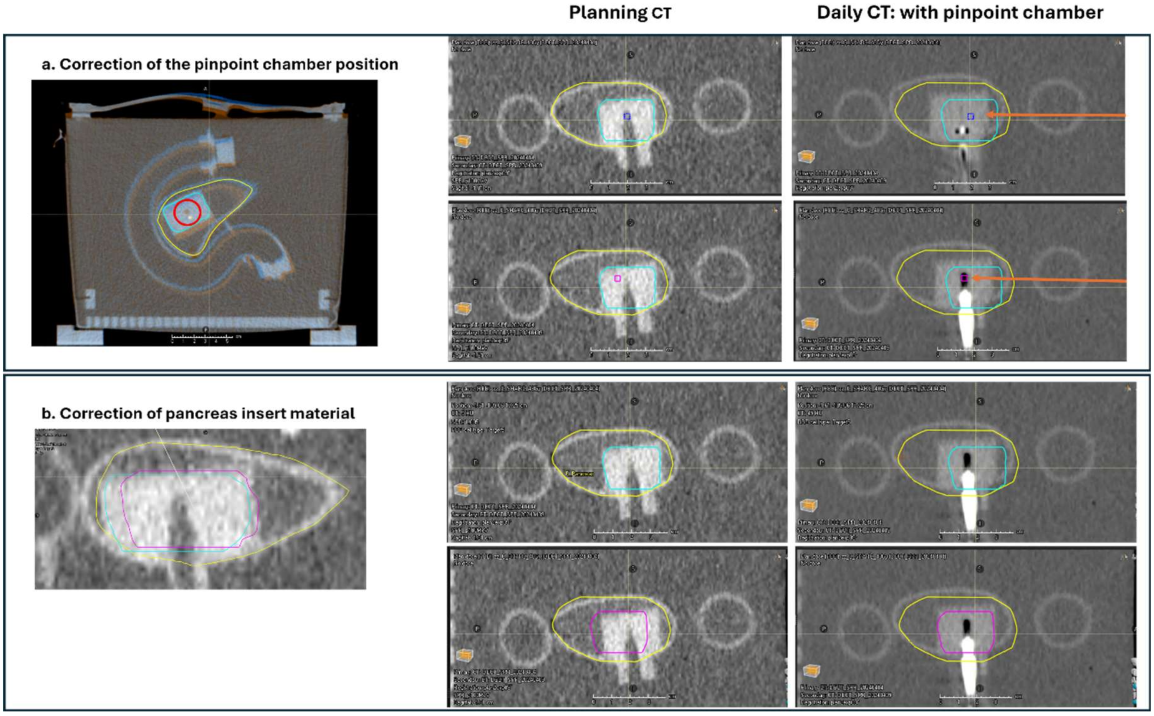

Figure S1.1: Corrections made to translate the measured results to the dose values computed by the Treatment Planning System (TPS), following registration of the daily CT to the planning CT. a. Correction of the position of the pinpoint chamber on the pancreas - on the left: overlay of daily CT (with orange counters) on the planning CT (in blue); on the right: differences between pinpoint chamber position in planning or the daily CT (dark blue and pink volumes, respectively). b. Correction of the position of the insert inside the pancreas - on the left: difference between insert definition based on the planning CT (blue) and daily CT (pink); on the right: CTs showing the definition of the insert in both CT's. Moreover, the pancreas volume, delineated based on the planning CT, is shown in yellow.

In addition to the planning CT, an additional CT was acquired on each irradiation day using the same image acquisition settings and reconstruction parameters, with the only difference being the inclusion of the pinpoint chamber in the daily CT (referred to as daily CT). A comparison between the daily and the planning CTs revealed deviations in the point of measurement, likely due to the weight of the pinpoint chamber on the 3D-printed organ, as shown in Figure S1.1.

Due to this, a two-step correction was applied to accurately compare the doses measured during each irradiation with the planned doses in the Treatment Planning System (TPS). First, we identified the pinpoint measurement location on the day of irradiation and then determined its corresponding position in the planning CT. This process is illustrated in Figure S1.1.a, which shows the old and new pinpoint chamber positions in light blue and pink, respectively. Additionally, the position of the higher density insert within the pancreas was corrected, as demonstrated in Figure S1.1.b. This insert was delineated on both the planning and daily CT scans (in blue and pink respectively). Subsequently, the region of the phantom corresponding to the insert in the planning CT was overwritten with insert-like material, equivalent to 1.170 g/cm^3. Conversely, the region of the phantom that should no longer contain the insert was overwritten with water-equivalent material matching the body-tissue equivalent mix that fills the phantom, at 1.040 g/cm^3.

S2. Dose distribution across different planning strategies

Figure S2.1. shows axial slices of the RBE-dose, physical dose, and LETd distributions of the physically optimized plans for the cylindrical (top) and the pancreas-shaped (bottom) PTV.

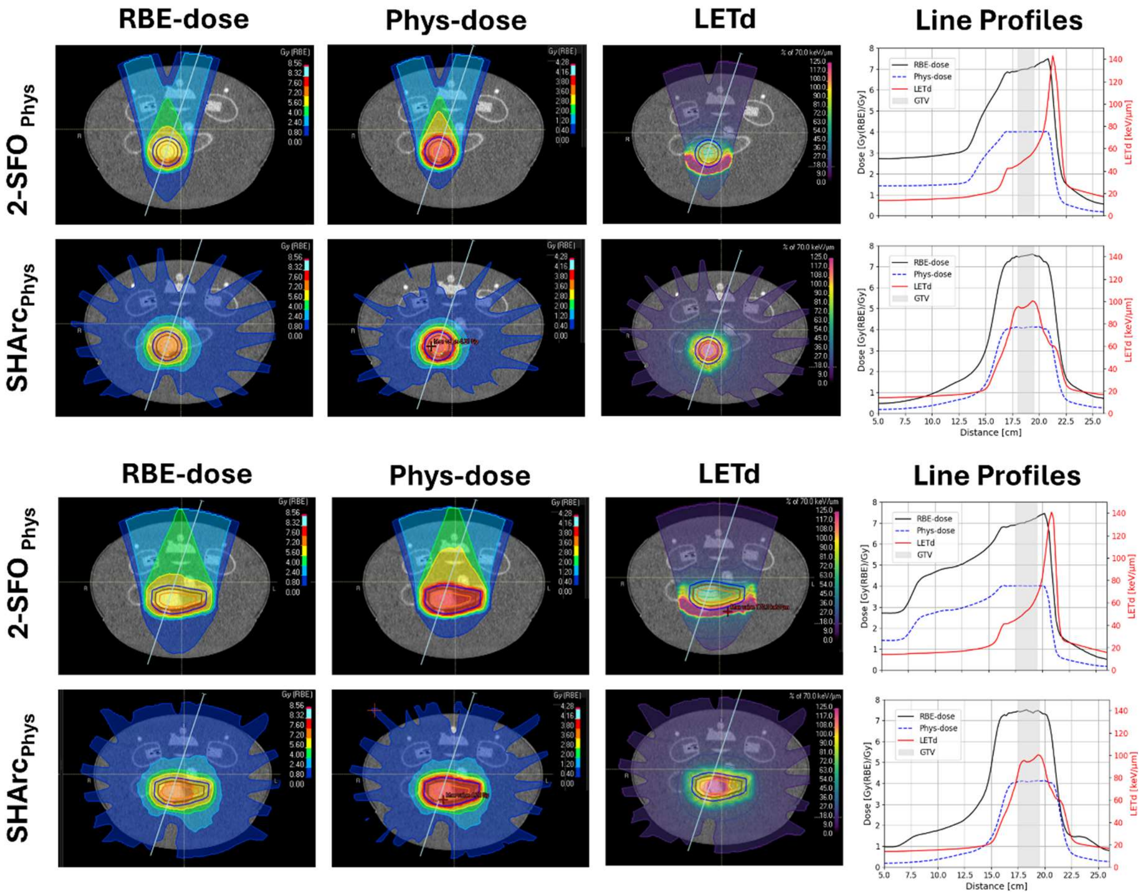

Figure S.2.1: Treatment planning strategies optimized considering only physical dose-based objectives, for (a) the cylindrical target and (b) the pancreas-shaped target. For each plan (line in the figure): axial slice of the relative biological effectiveness (RBE)-weighted dose and the physical dose distributions, as well as dose-averaged Linear Energy Transfer (LETd) distribution (left to right). Moreover, RBE-weighted and physical dose profiles (in black and blue), as well as LETd profiles (in red) across a line (in white) are represented as a function of penetrated depth (in cm).

Figure S2.2. shows physical dose profiles for a single beam across another axial slice comparing the three different beam configurations, with the profiles categorized by colors according optimization strategy.

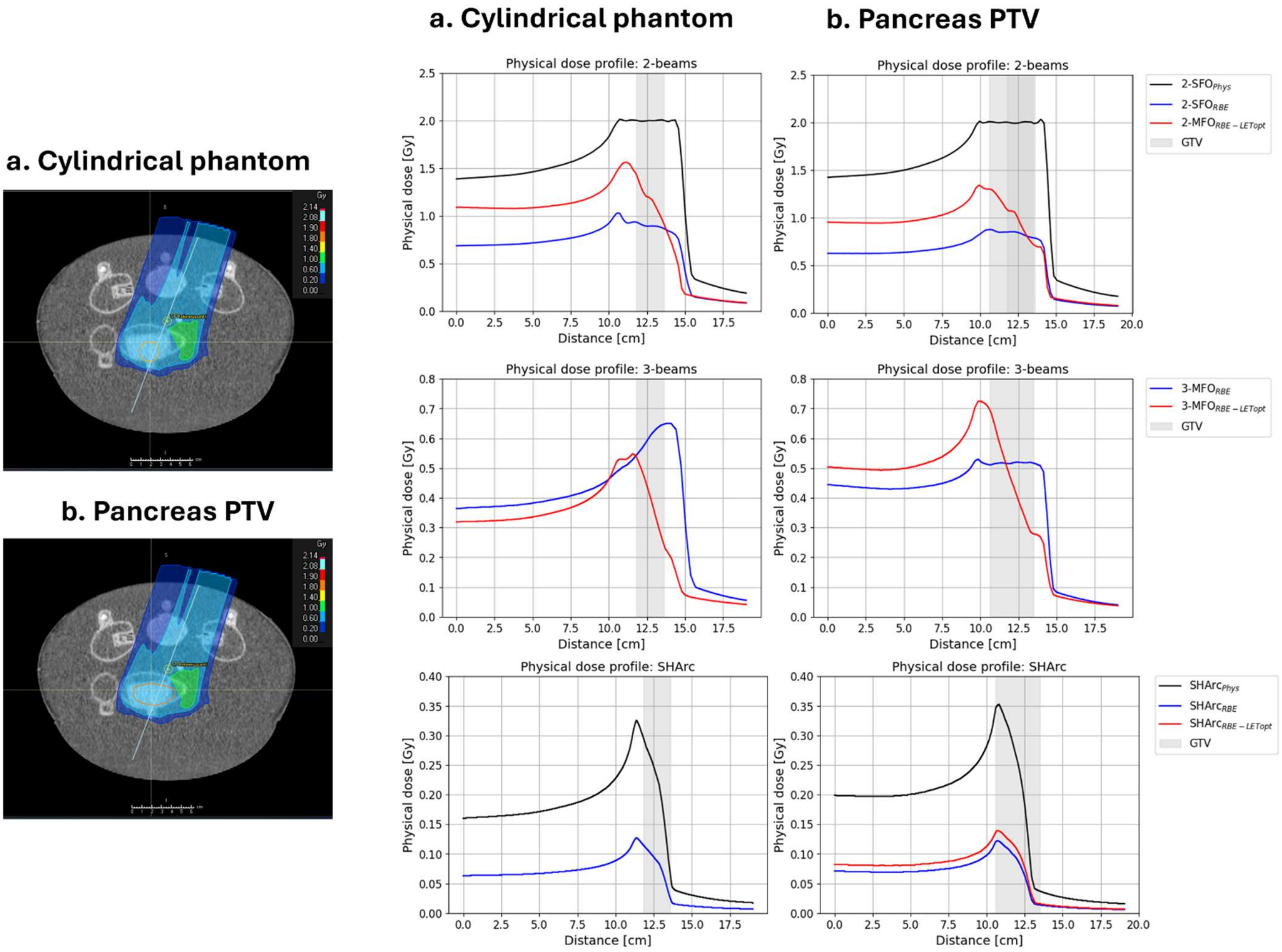

Figure S2.2: Comparison of physical-dose profiles for a single beam, for the different beam setups - 2-beam (top), 3-beam (middle), and Spot-Scanning Hadron Arc (SHArc) (bottom)- and optimization strategies, for (a) the cylindrical Planning Target Volume (PTV) and (b) pancreas PTV. On the left, two axial slices display the physical dose distribution for the 2-SFO_{RBE} plan for each PTV volume, as well as the line (in white) across which the dose profiles were retrieved. On the right, profiles for physically optimized plans are indicated by black lines, relative biological effectiveness (RBE)-optimized plans by blue lines, and dose-averaged Linear Energy Transfer (LETd)-optimized plans by red lines.

S3. Forward recalculations with SECT

The different plans which were initially optimized using the DECT image as planning CT, were recalculated based on the SECT image. Following this recalculation, the difference in percentage between DECT and SECT images, in terms of $D_{99\%}$ and the $D_{1\%}$ in the PTV, was computed and is presented in Table S3.1.

Table S3.1: Percentual difference in minimum and maximum target coverage, between the original relative biological effective (RBE)-dose computed based on the planning CT (DECT) and the respective forward calculation using the SECT performed on the same day/registration frame.

**Target**	**Plan**	**$\Delta D_{99\%}$ [%]** $\frac{D_{99\%\,|\,DECT} - D_{99\%\,|\,SECT}}{D_{99\%\,|\,DECT}}$x100	**$\Delta D_{1\%}$ [%]** $\frac{D_{1\%\,|\,DECT} - D_{1\%\,|\,SECT}}{D_{1\%\,|\,DECT}}$x100
Cylinder	2-SFO_{Phys}	0.00	-0.25
	2-SFO_{RBE}	0.00	0.74
	2-$MFO_{RBE\text{-}LETopt}$	0.26	1.21
	3-MFO_{RBE}	-0.25	0.00
	3-$MFO_{RBE\text{-}LETopt}$	0.26	1.47
	$SHArc_{Phys}$	1.79	4.10
	$SHArc_{RBE}$	1.02	2.68
Pancreas	2-SFO_{Phys}	0.00	-0.25
	2-SFO_{RBE}	0.00	0.49
	2-$MFO_{RBE\text{-}LETopt}$	-0.26	0.24
	3-MFO_{RBE}	-0.25	-0.25
	3-$MFO_{RBE\text{-}LETopt}$	0.51	0.98
	$SHArc_{Phys}$	1.53	3.38
	$SHArc_{RBE}$	0.77	2.69
	$SHArc_{RBE\text{-}LETopt}$	0.77	2.44

S4: Dosimetric measurements and evaluation

Following plan irradiation, the respective deviation to the TPS value was computed according to clinical quality assurance standards (Eq.1 in the Main Text). As there was no consistent pattern of the measured dose being higher or lower than expected, the absolute deviation between measured and expected doses was used. Moreover, the correction for the pinpoint chamber position was accounted for in this calculation. A summary of the mean measured values for each beam after three irradiations, for the 2-field and 3-field plans, along with the mean deviations from the TPS values, is presented in Tables S4.1-3.

Table S4.1-3: (1-2) Dosimetric measurements for Beams 1 and 2 in the 2- and 3-beam configurations, respectively, (3) Dosimetric measurements for Beam 3 in the 3-beam configuration. The tables include the expected physical dose (Gy) in the point of measurement calculated by the Treatment Planning System (TPS), the mean measured physical dose (Gy) with corresponding standard deviation (%), and the mean deviation (%) between measured and expected doses, along with the standard deviation for the deviations (%).

Table S4.1: Beam 1

**Target**	**Volume** [cm^3]	**Plan**	**TPS dose** [Gy]	**Measured dose** [Gy]	**Deviation to TPS** [%]
Cylinder	12.36	2-SFO$_{Phys}$	2.01	2.00 (±0.10%)	0.39 (±0.29%)
		2-SFO$_{RBE}$	0.94	0.94 (±0.32%)	0.18 (±0.15%)
		2-MFO$_{RBE\text{-}LETopt}$	0.87	0.90 (±3.35%)	2.28 (±1.13%)
		3-MFO$_{RBE}$	0.58	0.59 (±0.17%)	1.46 (±0.17%)
		3-MFO$_{RBE\text{-}LETopt}$	0.70	0.70 (±0.14%)	0.14 (±0.06%)
Pancreas	27.41	2-SFO$_{Phys}$	2.00	1.99 (±0.15%)	0.37 (±0.12%)
		2-SFO$_{RBE}$	0.93	0.95 (±0.74%)	1.47 (±0.60%)
		2-MFO$_{RBE\text{-}LETopt}$	0.92	0.96 (±0.94%)	1.48 (±0.47%)
		3-MFO$_{RBE}$	0.69	0.71 (±2.82%)	3.27 (±2.46%)
		3-MFO$_{RBE\text{-}LETopt}$	0.74	0.81 (±0.50%)	7.95 (±2.45%)

Table S4.2: Beam 2

**Target**	**Volume** [cm^3]	**Plan**	**TPS dose** [Gy]	**Measured dose** [Gy]	**Deviation to TPS** [%]
		2-SFO$_{Phys}$	2.00	1.99 (±0.25%)	0.57 (±0.25%)
		2-SFO$_{RBE}$	0.93	0.92 (±0.76%)	0.42 (±0.17%)

**Target**	**Volume** [cm^3]	**Plan**	**TPS dose** [Gy]	**Measured dose** [Gy]	**Deviation to TPS** [%]
Cylinder	12.36	2-$MFO_{RBE\text{-}LETopt}$	0.85	0.90 (±1.00%)	2.58 (±1.44%)
		3-MFO_{RBE}	0.69	0.59 (±0.17%)	1.00 (±0.15%)
		3-$MFO_{RBE\text{-}LETopt}$	0.71	0.70 (±0.14%)	0.87 (±0.15%)
Pancreas	27.41	2-SFO_{Phys}	2.00	1.99 (±0.15%)	0.44 (±0.00%)
		2-SFO_{RBE}	0.91	0.89 (±0.90%)	1.45 (±0.53%)
		2-$MFO_{RBE\text{-}LETopt}$	0.87	0.90 (±1.11%)	2.72 (±0.52%)
		3-MFO_{RBE}	0.38	0.35 (±1.43%)	2.69 (±0.10%)
		3-$MFO_{RBE\text{-}LETopt}$	0.44	0.45 (±1.56%)	2.63 (±1.46%)

Table S4.3: Beam 3

**Target**	**Volume** [cm^3]	**Plan**	**TPS dose** [Gy]	**Measured dose** [Gy]	**Deviation to TPS** [%]
Cylinder	12.36	3-MFO_{RBE}	0.67	0.66 (±0.15%)	0.78 (±0.13%)
		3-$MFO_{RBE\text{-}LETopt}$	0.32	0.39 (±0.00%)	5.18 (±0.03%)
Pancreas	27.41	3-MFO_{RBE}	0.79	0.78 (±2.30%)	0.91 (±0.90%)
		3-$MFO_{RBE\text{-}LETopt}$	0.54	0.55 (±2.17%)	2.15 (±0.99%)
